# Impact of health literacy and self-care behaviors on health-related quality of life in Iranians with type 2 diabetes: a cross-sectional study

**DOI:** 10.1186/s12955-020-01613-8

**Published:** 2020-11-04

**Authors:** Saber Gaffari-fam, Yosef Lotfi, Amin Daemi, Towhid Babazadeh, Ehsan Sarbazi, Ghader Dargahi-Abbasabad, Hamed Abri

**Affiliations:** 1 School of Nursing of Miandoab City, Urmia University of Medical Sciences, Urmia, Iran; 2 MSc in Nursing Education, Islamic Azad University, Sarab Branch, Sarab, Iran; 3 Health Management and Economics Research Center, Iran University of Medical Sciences, Tehran, Iran; 4 Department of Public Health, Sarab Faculty of Medical Science, Sarab, Iran; 5 Student Research Committee, Tabriz University of Medical Sciences, Tabriz, Iran; 6 MSc of Epidemiology, Research Center of Psychiatry and Behavioral Science, Razi Hospital, Tabriz University of Medical Science, Tabriz, Iran

**Keywords:** Health literacy, Self-care, Quality of life, Diabetes mellitus, Iran

## Abstract

**Background:**

Regarding the importance of health literacy as a key factor in self-care, appropriate understanding of health information by patients with type 2 diabetes mellitus (T2DM) is fundamental for better management of risk factors, which can also benefit their quality of life. This study aimed to describe the relationship between health literacy (HL), and self-care behaviors with health-related quality of life (HRQL) in patients with T2DM.

**Methods:**

A cross-sectional survey was done in Iran in 2019. Patients were recruited randomly from health centers by medical records (n = 192, 55.2% male, mean age 58.12 years). The data collection included demographic form, health literacy questionnaire, diabetes self-care behavior questionnaire, and world health organization’s Quality of Life-BREF (WHOQOL-BREF). Analyses were adjusted for confounders using hierarchical regression analysis.

**Results:**

HL as predictor variables explained 47.5% of variance in overall HRQL (*p* value < 0.001), reading health information was the strongest HL dimension (β = 0.478). Self-care behaviors explained an additional 13.6% of the HRQL variance. In total, 65.5% of the variation in the HRQL is explained by the HL, self-care behavior, and the demographic variables.

**Conclusion:**

We found that more almost two-third of the HRQL explained by the HL and self-care behaviors. Given the importance of health literacy and self-care behaviors in the quality of life in patients with T2DM, adoption of health-promoting behaviors and increasing health literacy can be beneficial for promoting quality of life among these patients.

## Introduction

Type 2 diabetes mellitus (T2DM) is a chronic disease that its related complications have reached epidemic levels, affecting the global economy and health [1, 2]. Diabetes caused 4 million deaths in 2017, and cost more than USD 727 billion for people with diabetes, which is equal to 12.5% of the overall healthcare budget worldwide [3]. According to the reports by the International Diabetes Federation, in 2017, 1 in 11 adults aged 20–79 years had T2DM worldwide [4]. As well, report of the World Health Organization (WHO) indicated that in 2016, diabetes was the 7th leading cause of death worldwide [5]. The number of people with diabetes in the Middle East and North Africa (MENA) region is predicted to be more than double by 2045 [6]. Iran ranks third in the MENA region after Egypt and Pakistan in terms of the total population of adults with diabetes (4396 diabetes cases (20–79) in 1000 [7]. Approximately 11.4% of the Iranian adult population has T2DM, and by 2030, about 9 million Iranians could be at risk of developing the disease [8].

T2DM is a major cause of blindness, kidney failure, heart attacks, stroke and amputation of the lower extremities [9]. To prevent serious morbidity and mortality, diabetes treatment requires dedication to rigorous self-care behaviors in many ways, including healthy food choices, physical activity, proper medications intake and control of blood glucose [10]. Although self-care behaviors are determinant in controlling the disease and its related complications, it is highly challenging because factors such as diabetic patient’s knowledge of the diabetes, physical skills, emotional factors, self-efficacy and other perceptions interact with and affect the self-care behavior [11]. Promoting self-care behaviors of diabetes, which is an important factor for metabolic regulation and the elimination of diabetes complications can lead to improvements in HRQL [12, 13]. Based on definition by the WHO, HRQL is “individual’s perception of the current situation with respect to the culture and value system in which he/she lives and their relationship with the individual’s goals, expectations, standards and priorities” [12]. HRQL is seen as the primary patient-centered outcome of healthcare and has increasingly been used in medical interventions and population health surveys as a comprehensive health indicator [14]. Quality of life is an important factor in diabetes because poor quality of life contributes to decreased self-care, which in turn leads to worsened glycemic control, increased risks of complications, and an overwhelming deterioration of diabetes in both the short run and the long run [15]. Diabetes dramatically raise the risk of developing blindness, end-stage renal disease, lower limb amputations, as well as increases mortality due to coronary artery disease, cerebrovascular disease, or peripheral vascular disease [16].

Some studies suggested that HL is effective in improving health outcomes such as self-care behaviors [17] and HRQL [18] among patients with diabetes. HL is a relatively new concept in health promotion research [19] and defined by Sorensen et al. (2012). as “people’s knowledge, motivation, and competences to access, understand, appraise, and apply health information in order to improve HRQL during the life course” [20]. Also, according to the WHO definition, “HL represents the cognitive and social skills which determine the motivation and ability of individuals to gain access to, understand, and use the information in ways which promote and maintain good health” [21]. HL requires people’s knowledge in order to make judgments and take decisions in everyday life concerning healthcare, disease prevention and health promotion to preserve or increase the HRQL over the life course. It may be more important than ever to have sufficient HL because people are expected to take part in health decisions and take responsibility for their own health despite more complicated health problems and the need to navigate a more complex health system [22].

The findings of a national study in the US demonstrated that over 33% of people lack sufficient HL [23]. In Iran, just 31% of patients with diabetes had sufficient HL [24]. Previous studies has associated low HL with poor HRQL in patients with chronic diseases [25] including T2DM [18]. The literature indicates that HL is an appropriate predictor for health outcome in patients [26], and low HL is associated with a wide variety of adverse complications on health care process and outcomes [27]. Also, optimal HL seems critical to improving HRQL and health status, particularly when targeted primary and secondary preventive strategies are struggling to achieve the expected rates [28, 29].

To address and to help bridge the gap between HL and health outcomes, particularly in the diabetic population, we conducted this study to explore the association between HL, self-care behaviors and HRQL among a population-based sample of adults with T2DM.

## Material

### Study setting and subjects

We conducted a cross-sectional study from September 2019 to February 2020 among T2DM patients in Sarab city, East Azerbaijan Province, Iran. East Azerbaijan Province is situated in northwestern Iran and has mostly foothill and mountainous areas with an area of around 45,490 square kilometers. The province is bounded in the north by the Republic of Azerbaijan and Armenia. Sarab is located east of the province and sided the province of Ardabil. Its capital, Sarab city is 636 km. from Tehran, 130 km. from Tabriz to the east of Bostan Abad. This township resting amongst the high mountains of Bozqoosh and Sahand.

Based on the test exam from the t-test family, and from the “correlation: point biserial model” in the statistical test, using the G*Power software, the input parameters for two-tail hypothesis, effect size = 0.245, α error probability = 0.05, power (1-β error probability = 0.95), respectively, the sample size determined as 205 [30]. Finally, 192 people agreed to participate in the study and all of them completed the written questionnaire (Response rate = 93.7%) to participate in the study.

### Data collection

Participants were recruited randomly from health centers by medical records among 6000 patients by using the command “=RANDBETWEEN(1,6000)” in the Excel software. The study sample was invited from amongst the people who met the study entry criteria. Subsequently, after referring to the healthcare centers and giving a complete explanation to the subjects and obtaining informed consent from them, they answered to the questionnaires in a consultation room. Besides filling the questionnaires, the participants interviewed by trained interviewers. The interviews lasted approximately for 25 min.

Inclusion criteria were diagnosis of T2DM in accordance with the final diagnosis instructions of the Iranian national laboratory standards, ≥ 30 years of age, with or without complications, lack of chronic illnesses and diabetes complications, and at least a six-month history of diabetes and having low education (secondary education and lower). The exclusion criteria were as follow: not having psychotic disorder, dementia, or blindness and refusal of participation and any medical problems that prevented self-care behaviors (such as exercise and regular physical activity) in the study.

### Study instruments and their validity and reliability

The instruments for collecting data included: (1) demographics form; (2) health literacy questionnaire; (3) diabetes self-care behavior questionnaire; (4) WHO quality of Life-BREF (WHOQOL-BREF).

### Demographics form

The demographics form consisted of gender (male, female), age (> 50, 50 ≤), job (employed, unemployed, housewife), marital status (married or unmarried).

### Health Literacy Questionnaire (HLQ)

To assess HL was used a valid and reliable tool which was established by Montazeri et al. [31]. A full explanation of the HLQ and its five subscales is described as follow:

1. *Reading health information* the four-item subscale was evaluated on a five-point Likert-type scale ranging from 1 to 5 (1 = completely difficult through 5 = completely easy). An example of this aspect was: “reading health education materials (e.g., booklet, pamphlet, and educational brochures) was easy for me”. Cronbach’s alpha for this subscale was measured 0.72. The total possible scores varied from 4 to 20. The higher the score, the greater the reading abilities.
2. *Understanding health information* was assessed using seven items (e.g., “I can acquire the required health and medical information from various sources”). Each item was scored on a 5-point scale from 1 to 5 (1 = completely difficult through 5 = completely easy). For this subscale, Cronbach’s alpha was 0.86. The theoretical range for this subscale was 7–35 and the higher the ratings, the more comprehensible to understand health information.
3. *Appraisal of health information* was assessed applying four items. (e.g., “I can get information about healthy nutrition”). Each item was scored on a five-point Likert-type scale that ranged from 1 (never) to 5 (always). Cronbach’s alpha for this subscale was 0.77. The total score on this subscale could range from 4 to 20. A high total score shows a high ability of appraisal of health information.
4. *Ability to access health information* was measured by six items (e.g., “I can obtain information about my illness”). A five-point Likert-type scale was used (always = 5, most of the time = 4, sometimes = 3, seldom = 2 and never = 1). Cronbach’s alpha for this subscale was 0.86. The total possible scores ranged from 6 to 30, higher the score, the more ability to access health information.
5. *Decision making* was a twelve-item subscale designed to measure the ability to decide health-related behaviors. Sample of items is: “I avoid doing things or taking materials that might increase my weight” even if the symptoms of the disease would be disappeared. The items were scored on a five-point Likert-type scale ranging from 1 to 5 (always = 5, most of the time = 4, sometimes = 3, seldom = 2 and never = 1). For this subscale, Cronbach’s alpha was 0.89. The higher the ranking, the better decision making was concluded.

### Diabetes self-care behavior questionnaire

We used a 12-item summary of diabetes self-care activities scale, [32] to measure self-care performances. This questionnaire had been validated by Didarloo et al., among people with type 2 diabetes in Iran [33]. The Cronbach’s alpha was assessed 0.74. The scale measures frequency of self-care behaviors in the last 7 days in four dimensions of the diet (6 items), glucose testing (2 items), medications (2 items) and physical activity (2 items). The total self-care activities score on this index may range from 0 to 84 in which higher scores indicate higher self-care behaviors adopted by the patients.

### WHO Quality of Life-BREF (WHOQOL-BREF) questionnaire

A standardized Persian version of the WHO’s questionnaire (WHOQOL-BREF) was applied to assess HRQL among participants. Validity and reliability of this translation of WHOQOL-BREF was approved in a study conducted by Nejat et al. [34] in Iran. This instrument includes four domains: physical health (7 items; α = 0.70), mental health (6 items; α = 0.73), social relationships (3 items; α = 0.55), and environmental health (8 items; α = 0.84). The items were rated on a five-item Likert scale ranged from 1 to 5 for all the domains. The scoring of the answers to items 3, 4 and 26 has been reversed. A score of 0 to 100 has been obtained after performing the calculations at each domain. The ranking of 0 shows the poorest quality of life and the best condition calculated as 100. Such values can also be translated to a ranking of 4 to 20 [35]. In this study the scores range from 0 to 100 in each dimension.

### Statistical analysis

For the categorical variables, percentages and frequencies applied while for the continuous variables, the mean and standard deviation or median and quartile deviation, depending on the distribution of the data. All analyses were conducted using SPSS 21.

Bivariate comparisons were performed using the independent samples T-test for quantitative variables. Pearson Correlation are used in to measure a relationship is between between HL and self-care with HRQL. Pearson's r can range from − 1 to 1.

Hierarchical regression analysis for HRQL was carried out by entering 3 separate blocks of independent variables. Block 1 consisted of age, gender, job, marital status which were operationally defined as the demographic characteristics and were entered first. Block 2 included HL dimensions. Block 3 was consisted of the scores from demographic characteristics, HL, self-care behaviors. After the entry of each block, we measured the adjusted *R*2 change to decide the proportion of variance described by HRQL. Tests for multi-colinearity, normality, and influential data points demonstrated that the assumptions of regressions were met. The significance level was set to α = 0.05.

## Results

Table 1 demonstrates the demographics of the participants . The mean age of participants was 58.12 years (SD = 11.83) and among the 192 T2DM, 72.4% of them were in age groups of 50 and higher, over half of the samples were male (55.2%), and also, only 19.8% of the patients were unemployed. Most participants were married (82.3%) (Table 1).

**Table 1:** Relationship between HRQL and some of demographic characteristics among participants

Variables	F (%)	PH*	*p* value	MH*	*p* value	SR *	*p* value	EH*	*p* value
		Mean (SD)		Mean (SD)		Mean (SD)		Mean (SD)	
Age groups (years)**									
50 >	53 (27.6)	51.54 (13.02)	0.037	50.20 (10.54)	0.082	51.90 (16.91)	0.016	51.64 (14.07)	0.011
50 ≤	139 (72.4)	47.17 (13.44)		46.80 (12.02)		47.15 (6.55)		45.65 (14.16)	
Gender**									
Male	106 (55.2)	48.83 (13.72)	0.624	47.68 (11.75)	0.984	48.69 (17.99)	0.443	48.01 (14.78)	0.487
Female	86 (44.8)	47.82 (13.12)		47.81 (11.70)		48.13 (15.17)		46.45 (13.85)	
Job^#^									
Employed	77 (40.1)	50.24 (13.91)	0.067	49.83 (11.56)	0.030	50.43 (16.96)	0.025	50.41 (14.15)	0.009
Unemployed	38 (19.8)	44.13 (13.25)		43.42 (11.44)		41.76 (19.45)		41.94 (16.10)	
Housewife	77 (40.1)	48.61 (12.72)		47.79 (11.53)		49.77 (14.30)		46.84 (12.94)	
Marital status**									
Married	158 (82.3)	49.01 (13.57)	0.206	48.41 (11.41)	0.059	49.34 (16.49)	0.088	48.06 (13.91)	0.086
Unmarried	34 (17.7)	45.47 (12.56)		44.61 (12.66)		44.29 (17.51)		43.76 (15.99)	

^*^PH, physical health; MH, mental health; SH, social relationships; EH, environmental health

^**^p value based t-independent exam

^#^*p* value based one-way ANOVA exam

### Health-related quality of life

The mean score of patients’ quality of life in dimensions of physical health was 48.32 ± 13.43, mental health 47.74 ± 11.70, social relationships 48.44 ± 16.74 and environmental health 47.30 ± 14.35.

In terms of age groups, significant differences were observed in all aspects of quality of life of the patients (*p* value < 0.05), except for mental health dimension. There was significant difference between physical health and the age group (*p* value = 0.037); mental health and the job (*p* value = 0.030); social relationships and the age groups (*p* value = 0.016) and the job (*p* value = 0.025); environmental health and the age groups (*p* value = 0.011) and the job (*p* value = 0.009) (Table 1).

As it can be seen in Table 2, all health literacy dimensions and self-care behaviors except medication domain were positively correlated with the overall HRQL (*p* value ≤ 0.05) (Table 2). Descriptive statistics of the measured variables are demonstrated in Table 3, the highest score belonged to the decision-making and the lowest dimension was reading health information.

**Table 2:** Bivariate correlation matrix of the relationship between HL and self-care with HRQL

Variables	1	2	3	4	5	6	7	8	9	10
1. Reading health information	1									
2. Ability to access health information	0.688*	1								
3. Understanding health information	0.725*	0.747*	1							
4. Appraisal of health information	0.618*	0.746*	0.680*	1						
5. Decision-making	0.386*	0.732*	0.647*	0.520*	1					
6. Diet	0.123	0.141	0.175*	0.254*	0.473*	1				
7. Physical activity	0.171*	0.207*	0.174*	0.255*	0.192*	0.348	1			
8. Glucose testing	0.391*	0.142	0.238*	0.158*	0.103	0.032	0.358*			
9. Medications	0.106	0.055	0.083	0.071	0.146*	0.187*	0.205*	0.086	1	
10. Quality of Life	0.207*	0.488*	0.452*	0.531*	0.562*	0.542*	0.368*	0.174*	0.010	1

^*^Correlation is significant at the 0.05 level (two-tailed)

**Table 3:** Distribution of health literacy scores and dimensions of it’s and self-care behavior

Variables	Mean (± SD)
HL	86.66 (± 20.6)
Self-care behaviors	43.71 (± 10.11)
Reading health information	7.48 (± 4.10)
Ability to access health information	15.02 (± 5.35)
Understanding health information	17.98 (± 5.11)
Appraisal of health information	10.51 (± 3.08)
Decision-making	35.65 (± 7.28)

### Predictors of HRQL: continuous modeling

In the hierarchical regression model examining effects of demographic characteristics, health literacy, self-care behaviors on overall HRQL. In the step 1, demographic characteristics accounted for 4.6% of the variation in overall HRQL (F = 2.22; *p* value = 0.068), that is, approximately 4.6% of the variation in overall HRQL is explained by the demographic variables. Table 3 displays that age (ß = − 0.156; *p* value = 0.010) was statistically associated with better overall HRQL. HL dimensions as predictor variables (step 2) explained an additional 47.5% of variation in overall HRQL (F = 35.69; *p* value < 0.001). In step 3, self-care behaviors were added, which explained an additional 13.6% of the variation (F = 17.39; *p* value < 0.001). In total; demographic characteristics, HL dimensions and self-care behavior were able to explain 65.5% of the variation in overall HRQL (Table 4).

**Table 4:** Hierarchical linear regression for prediction HRQL through demographic characteristics, self-care behaviors and health literacy

Variables	ß	R2 change	F change	SE	*p* value
Step 1					
Age	− 0.156*	0.046	2.22	8.17	0.068
Gender	0.033			13.98	
Job	0.074			7.78	
Marriage	0.117			9.26	
Step 2					
Age	− 0.124*	0.475	35.69	6.16	0.001
Gender	0.001			10.31	
Job	0.059			5.80	
Marriage	0.106*			6.70	
Reading health information	0.478*			0.96	
Ability to access health information	0.372*			0.87	
Understanding health information	0.089			0.99	
Appraisal of health information	0.266*			1.35	
Decision-making	0.380*			0.48	
Step 3					
Age	− 0.126*	0.136	17.39	5.42	0.001
Gender	0.074			8.98	
Job	0.040			5.07	
Marriage	0.113*			5.84	
Reading health information	0.372*			0.96	
Ability to access health information	0.364*			0.76	
Understanding health information	0.088			0.85	
Appraisal of health information	0.148			1.18	
Decision-making	0.208*			0.46	
Diet	0.341*			0.39	
Exercise	0.126*			0.78	
Glucose monitoring	0.143*			1.44	
Medications	0.081			2.37	
Total R2	–	0.655	–	–	
Adjusted R2	–	0.630	–	–	

*p < 0.05

## Discussion

HRQL is one of the important outcomes used to evaluate the effect of management of chronic diseases on health, and reflects the physical and psychosocial burden of a particular disease. Lower HL and self-care were associated with worse HRQL even after adjusting for potential confounders.

In the present study, the mean HRQL score found to be 49.14, which was similar to another study conducted in the Chaldoran city, West Azerbaijan, Iran (2017) that used the same measurement among patients with T2DM [36], and indicates that the patients with T2DM had a poor HRQL in both studies. In this study, also, HRQL of the participants was lower in domains of mental health and environmental health. This findings is consistent with studies conducted by Babazadeh et al. (2017) [36] in Iran and Wang et al. (2001) [37] in China on patients with T2DM. One possible reason for the poor quality of life of patients in the mental health domain may be the effect of physical disorders on psychological health.

A significant association was found between age groups and domains of the HRQL except mental health. Similar to our finding, another study showed that with increasing the patients’ age, their HRQL significantly decreased [38]. In another study conducted by Lindsay et al. (2011), the HRQL score declined with increasing age [39]. Also, in Glasgow et al. (1997) study indicated that younger patients had a better HRQL [40]. As the age increases, the risk of developing other diseases and the complications of diabetes will also increase.

In the current study, we found that having a job has positive effect on HRQL which is consistent with a prior study in Romania [41]. Having a job might be associated with a better economic situation and on the other hand, having one’s own income is likely to be related to having financial advantages and more welfare.

Similar to the findings of this study, Zareipour et al. [42] found a statistically significant association between HRQL and marital status of the patients. Rikard et al. (2016) believe that marriage as a social resource can improve one’s HL skills and subsequently prolong one’s lifespan [43]. According to the literature, marriage can lead to reinforcement of social communication and increase of support networks, as well as economic support for the payment for healthcare [44].

In this study, statistically significant positive association was observed between HL dimensions and the HRQL. Moreover, HL dimensions are able to predict 47.5% of the total variation in HRQL. In this regard, controversial findings have been reported in various studies. For example, in a study by Couture et al. [45] no association was found between HL and the HRQL, while one systematic review study demonstrated that higher HL leads to significant improvement in T2DM-related HRQL [46]. A meta-analysis on this relationship would be helpful for further research. The finding of this study means that people with low HL may pay low attention to their health status and thus have unhealthy behavioral patterns that have contributed a decline of HRQL [47]. The study of Sayah et al. (2016) in Alberta, Canada. also, revealed that low HL can lead to worse HRQL among adults with T2DM [18]. In another study, Lee et al. in China indicated that HL has direct positive effect on HRQL [48]. In addition, conducted studies in chronic disorders have reported that HL is an important predictor of health outcomes, like complications and HRQL [49]. Among the HL dimensions, reading health information was most important predictor of HRQL.

Text understanding is a process by which the reader goes through interaction and exposure to written language and at the same time it means extraction of concepts. The ability to understand is a mental process that people use to value their written forms and, with this skill, they extract meanings of different texts. Thus, the cause of obtaining these results is that understanding is mostly under the influence of internal factors [50]. Given the perceived challenges of inadequate HL in the self-management skills of diabetes, patients with inadequate HL are more likely to have problems in following the instructions of the care providers, reading prescription labels, and comprehending patient education materials [51]. These findings recommend that interventions should focus on promoting HL, especially increasing the ability to read health labels, in order to improve health outcomes like HRQL among patients with diabetes.

Our study presented that self-care behaviors has statistically significant positive correlation with HRQL. Also, self-care behaviors as predictor variables explained an additional 13.6% of variation in HRQL. A systematic review of clinical trials involving hospital discharge interventions showed that self-care in a community-based sample of older adults with chronic care needs results in less re-admission to the hospital and reduction in negative consequences [52]. Our study adds to these positive findings. Lee et al. [48] indicated that self-care activities are crucial to the link between HL and HRQL. Shi et al. [53] showed that HL was associated with blood glucose self-monitoring behaviors. HL impacts the health outcomes, and the pathway linking HL to HRQL is likely behavioral in nature, i.e., through disease self-management and self-care provided through clinicians. Similar with those found in previous study [36], of the self-car aspects, diet was strongest predictor of quality of life. In another study conducted in Iran on patients with diabetes, the researchers reported that nutritional performance can be effective to improve quality of life [54]. Therefore, interventions aiming at increasing the HRQL of diabetic patients should be taken in to account the nutrition and education of these patients for self-care.

## Limitations

The data are cross-sectional and therefore a causal association between health literacy and HRQL cannot be inferred. Responses to the questions of health literacy, HRQL, and socioeconomic factors are based on self-reporting and could be subject to social desirability, nonresponse, and other sources of bias. Our findings should therefore be replicated in the future with other quality of life instruments that require even lower literacy levels. We examined a population of subjects with low education (secondary education and lower) in Sarab city, East Azerbaijan province, which may not represent the population of adults with T2DM. One of the reasons for preferring people with low education was that the factors influencing their self-care actions might different from those with higher education. Also, in this study, appraisal of health information and understanding health information were not significant predictors of HRQL that one of the possible reasons could be low health literacy of participants. Majority of the participants in this sample were 50 years of age or older. One of the possible reasons for the participation of older people can the increased prevalence of diabetes with age.

## Conclusion

Our findings revealed that the HRQL of the T2DM patients, especially in both mental and environmental domain, is low. This finding recommends the importance of considering mental health related quality of life and environmental when developing interventional programs. All of the HL dimensions, except understanding health information and appraisal of health information, influence the HRQL in T2DM patients. Although, it seems that diet and ability to read health information be the most important factors of HRQL in T2DM patients. Interventions aimed at improving the diet and increasing ability to read among patients with T2DM is urgently needed. In total, more than half of the variation of HRQL was explained by the HL and self-care behaviors. Thus, adoption of health behaviors and increasing the HL can be best practices for improving the HRQL among T2DM patients.

## Data Availability

The dataset used for this research will be available from the corresponding author upon reasonable request.
